# Rapid electrochemical detection of single influenza viruses tagged with silver nanoparticles†
†Electronic supplementary information (ESI) available. See DOI: 10.1039/c6sc00412a


**DOI:** 10.1039/c6sc00412a

**Published:** 2016-02-25

**Authors:** Lior Sepunaru, Blake J. Plowman, Stanislav V. Sokolov, Neil P. Young, Richard G. Compton

**Affiliations:** a Department of Chemistry , Physical and Theoretical Chemistry Laboratory , Oxford University , Oxford OX1 3QZ , UK . Email: richard.compton@chem.ox.ac.uk; b Department of Materials , Oxford University , Oxford OX1 3PH , UK

## Abstract

Using a state of the art nano-electrochemical technique, we show that a single virus ‘tagged’ with silver nanoparticles can be rapidly detected in real time at the single virus level. A solution containing a low concentration of influenza virus is exposed to silver nanoparticles which are adsorbed onto the virus surface, as revealed by UV-Vis spectroscopy and transmission electron microscopy. With sufficient potential applied to a carbon electrode introduced into the solution, current spikes are observed which correspond to the oxidation of the nanoparticles decorating the virus. The frequency of the current spikes and their magnitude are linearly proportional to the virus concentration and to the surface coverage of the nanoparticles, respectively. Differences observed from single bacterium detection are discussed and a comparison with existing detection methods is made, with emphasis on the favourability of the proposed technique towards the realization of point of care test devices.

## Abstract 2

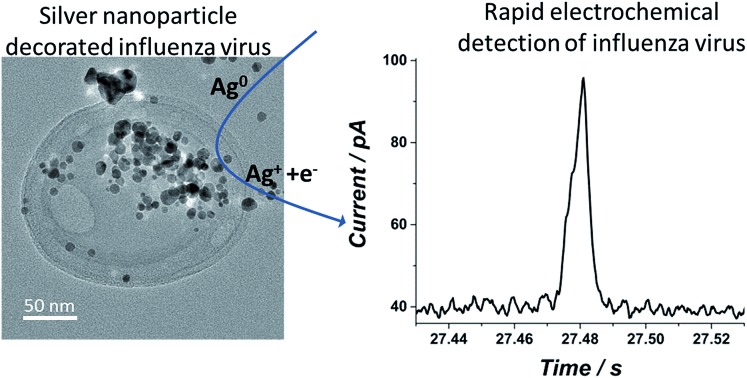
Using a state of the art nano-electrochemical technique, we show that a single virus ‘tagged’ with silver nanoparticles can be rapidly detected in real time at the single virus level.

## Introduction

The emerging need for rapid, reliable and cost effective methods of virus detection has been the subject of intensive investigation in the last decade.^1^–^8^ The need to replace the conventional cultivation of viruses in cell cultures has been addressed repeatedly and new methodologies have been sought. The vast majority of these can be divided into three major categories: the polymerase chain reaction (PCR),^9^,^10^ optical detection^11^–^13^ and electrical/electrochemical detection.^7^,^14^ At the moment the most promising directions for establishing a new generation of rapid viral detection methods rely frequently on real time (reverse transcriptase) PCR, immunosorbent assays or RNA amplification and detection.^15^–^17^ However, lately a new generation of sensors has emerged which incorporates nanoparticles as part of the sensor, mostly but not exclusively as the recognition element.^18^–^20^ Such use of nanoparticles has been shown to provide significant enhancement in the detection of the viruses. The integration of nanoparticles as part of the sensor provides or upgrades the above techniques with an increasing sensitivity and in some cases sensitivity. A further advantage of this strategy is the scope to create miniaturized and automated sensors through the use of electrochemical devices and pave the way towards point of care test devices.

In practice, the evaluation of a patient’s diagnosis is generally performed rapidly by an authorized person, which is usually heavily reliant on the patient's symptoms and the prevalence of a specific common disease/infection within the community at the time and location. This diagnosis by itself in many cases causes an unnecessary use of antibiotics. Moreover, the dosages of antibiotics or antiviral drugs given are uncorrelated with the actual intensity of the infection, as the magnitude of the infection is not quantified (*i.e.*, concentration of the bacteria or virus in the blood or urine). Therefore, there is a practical need for a simple, cost effective, point of care test device that can selectively detect in real time the existence of bacteria or a virus, and the *quantification* of the species' concentration is desirable. In this work we present a proof of concept towards this goal and show that by using commercially available electrochemical tools, the detection of individual viruses can be rapidly achieved. The method presented here exploits the interaction of viruses such as influenza with silver nanoparticles. This phenomena is extremely well investigated from the antiviral perspective,^21^ but can, as in this case, be used for low viral concentration detection due to the inherent electrochemical activity of the nanoparticle-modified virus surface upon interaction in solution. Moreover, we show that the virus concentration can be rapidly quantified and that the electrochemical signal detected can be distinguished from a signal arising from a single bacterium simply due to their different physical dimensions (number of nanoparticles covering the species).

## Results and discussion

In the following sections we describe steps towards the electrochemical detection of a single influenza virus ‘tagged’ with silver nanoparticles. The establishment of an intimate adsorption of silver nanoparticles on the virus surface is first shown by UV-Vis spectroscopy and transmission electron microscopy (TEM). Later, we verify that the virus covered with silver nanoparticles is electroactive, and we conclude with a realization of single virus electrochemical detection *via* the ‘nano-impacts’ technique together with a comparison to alternative techniques.

### Optical properties and characterization of silver nanoparticles in influenza virus media

**Fig. 1:**
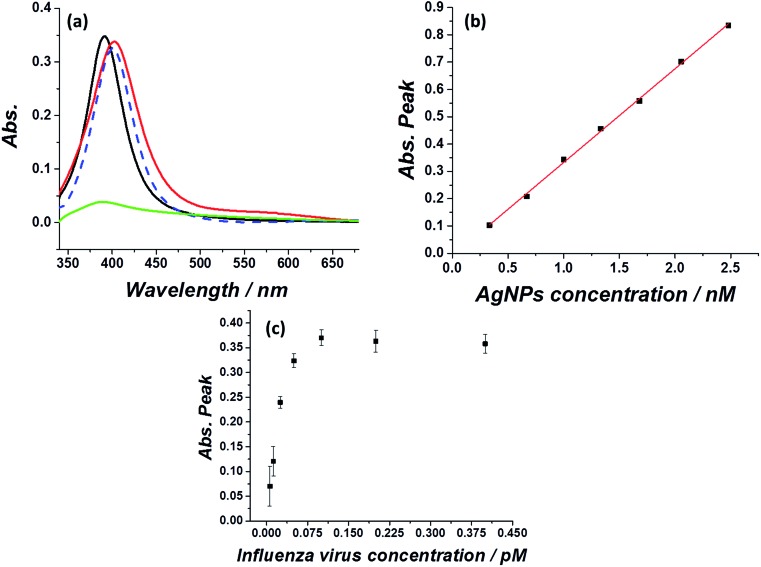
(a) UV-Vis absorption of a solution containing 1 nM silver nanoparticles in water (black), or 0.1 M KCl (green), with ∼1 pM influenza virus (red) or ∼1 pM *E. coli* (blue dashed) in the presence of 0.1 M KCl. (b) Linear correlation between the absorption peak at 401 nm and the amount of silver nanoparticles injected into a medium containing 1 pM of influenza virus. (c) Titration curve of the absorption signal at 401 nm arising from 1 nM silver nanoparticles injected into a solution containing different influenza virus concentrations.

First, optical and microscopic characterizations of the synthesized silver nanoparticles were made (see methods section for silver nanoparticles synthesis). As can be seen in Fig. 1a, the citrate capped silver nanoparticles show a typical plasmonic peak at 393 nm in water suspension (black curve), in agreement with previous reports.^22^,^23^ Exposing the silver nanoparticles to a medium of physiological ionic strength such as 0.1 M KCl immediately quenched the plasmonic peak (green curve), which is ascribed to agglomeration/aggregation of the nanoparticles.^24^ Next, the adherence and stabilization of the silver nanoparticles in an influenza virus medium was examined (influenza virus A, H1N1) by monitoring the UV-Vis spectra of the silver nanoparticles injected into a solution containing 0.1 M KCl and various concentrations of influenza virus. Fig. 1 further shows that the plasmonic peak of the silver nanoparticles was retained if the high ionic strength medium contained a sufficient concentration of influenza virus (red curve), with a minor red shift in the absorption peak to 401 nm. Similar results are seen for the exposure of silver nanoparticles to an *E. coli* solution (blue dashed curve).^25^ A comparison of the *E. coli* containing solution and influenza virus containing solution indicates that in both cases a similar red shift in the plasmonic peak is observed, pointing to a similar adsorption and stabilization mechanism. It should be noted however, that the absorption signals in these cases are indistinguishable, highlighting that the selective detection of the virus and bacteria cannot be achieved solely through such an optical method.

The magnitude of the silver nanoparticle absorption peak in the presence of influenza virus was then studied as a function of the nanoparticle concentration. The peak of the absorption signal increased linearly with the nanoparticle concentration (Fig. 1b). However, this effect is seen only when the virus/silver nanoparticles molarity ratio is below a certain threshold. The concentration of and size of the influenza virus in solution was first determined *via* nanoparticle tracking analysis (see Fig. S1†). Next, a 1 nM solution of silver nanoparticles was injected into a solution containing various concentrations of the virus and the plasmonic peak at 401 nm was monitored. From Fig. 1c it is seen that 0.125 pM influenza virus can stabilize approximately 1 nM silver nanoparticles. Above this molarity ratio (>1 : 8500), the virus is saturated with silver nanoparticles and can no longer stabilize additional silver nanoparticles injected into a 0.1 M KCl solution. This consequently causes quenching of plasmonic peak of the silver nanoparticles, as in the case of a solution absent of virus (for the full UV-Vis spectra see Fig. S2†). When the molarity ratio of influenza virus per nanoparticles was <1 : 8500, the plasmonic peak magnitude was constant, reflecting that the virus is not saturated with silver nanoparticles. This also indicates that the stabilization of silver nanoparticles by the virus is dominant to the natural agglomeration process occurring at this high ionic strength.

**Fig. 2:**
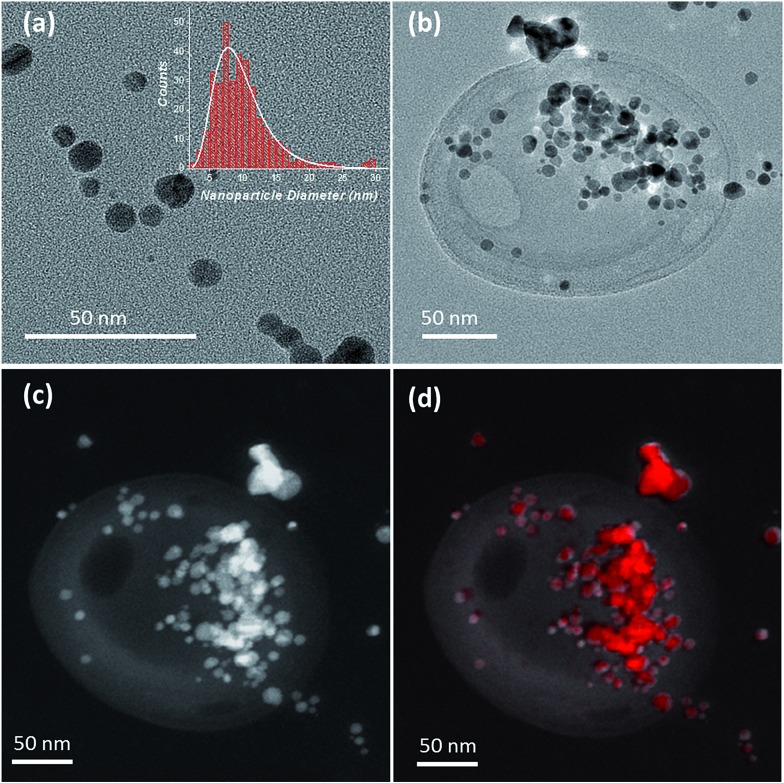
(a) TEM image of the silver nanoparticles (inset: size distribution of the silver nanoparticles). (b) Bright field TEM image of an influenza virus decorated with silver nanoparticles. A 4 μL solution of ∼5 pM influenza virus together with 20 nM silver nanoparticles in 0.1 M KCl was drop cast on a carbon grid and dried under ambient conditions. (c) Dark field TEM image of an influenza virus decorated with silver nanoparticles. (d) HAADF-STEM image of the area overlaid with spatially resolved EDX spectroscopy map of Ag Lα X-rays showing the virus decorated with silver nanoparticles (with silver shown in red).

The adsorption of the silver nanoparticles on the influenza virus surfaces was also confirmed by TEM imaging. As can be seen from Fig. 2a, the size of the silver nanoparticles showed a log distribution with a mean diameter of 9.1 nm and a multiplicative standard deviation of 0.4 nm (determined from the analysis of 345 nanoparticles, Fig. 2a inset). After incubation of the nanoparticles for 15 min in a solution containing the influenza virus in 0.1 M KCl (see Materials and methods), a clear localization of the nanoparticles on the virus is seen in Fig. 2b, c (for TEM characterization of the virus without silver nanoparticles see Fig. S3†). The silver content arising from the silver nanoparticles on the virus was also confirmed by energy dispersive X-ray (EDX) mapping and the results are shown in Fig. 2d and S3.† From the TEM image, it may seem that the silver nanoparticles are localized on a specific region of the virus. However, under ultra-high vacuum conditions the silver nanoparticles may appear bunched and a more detailed study is needed. We refer the reader to a few proposed adsorption mechanisms in ref. 26 and 27.

### *In vitro* modification of a carbon electrode with electroactive influenza virus

**Fig. 3:**
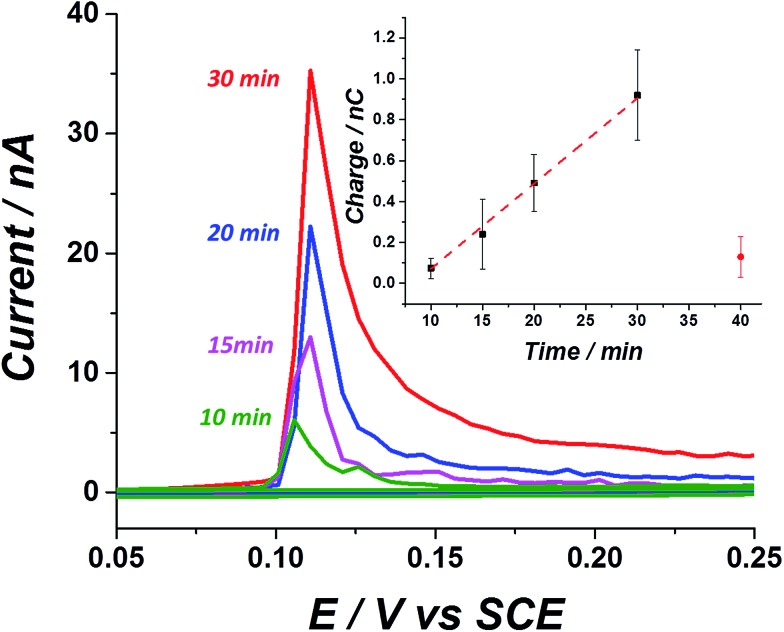
*In vitro* stripping voltammetric curves of a carbon electrode exposed to a solution of 1.26 pM influenza virus covered with silver nanoparticles at different designated immersion times. (Inset) linear relation between the total stripping charge as a function of immersion time at open circuit potential. The red circle corresponds to the stripping charge observed in a solution containing silver nanoparticles only, after 40 min exposure to an open circuit potential. A silver nanoparticle concentration of 8 nM was used for all the solutions.

After confirming the stabilization of silver nanoparticles in a virus containing medium, we next examined the electrochemical response arising from a solution containing influenza virus and the effect of exposure to silver nanoparticles. At first, the ‘sticking’ property of the virus was investigated using a carbon fibre wire, serving as a working electrode. Previous studies have shown that silver nanoparticles do not adsorb on a carbon electrode efficiently at an open circuit potential.^28^ On the other hand, it is well established that viruses may be readily adsorbed onto a variety of surfaces.^29^ This ‘sticky’ property of the influenza virus is manifested by a time dependent stripped charge seen in Fig. 3, after exposing the carbon wire for different times to a solution containing the influenza virus and silver nanoparticles. The longer the exposure time of the electrode to the solution at open circuit potential, the higher the observed stripped charge that is seen. A solution containing 1.26 pM influenza virus and 8 nM silver nanoparticles transforms the virus shell to an electrochemically active material. The oxidation potential of the silver nanoparticles in a KCl solution is around 0.1 V *vs.* SCE, and reflects the electrochemical transformation of the silver nanoparticles into silver chloride.^30^ Hence, by linearly ramping the electrode potential from 0.05 V to 0.25 V *vs.* SCE, the electrochemical process can be derived and the silver nanoparticles can be stripped from the electrode. Stripping voltammetry of the ‘tagged’ virus for different exposure times at open circuit potential reveals a linear increase in the magnitude of the stripping charge (Fig. 3 inset). The charge stripped reflects the oxidation of the virus surface modified with silver nanoparticles. The error associated with the stripped charge arises mostly from deviation in the electrochemically active area among the carbon wires used and also due to the stochastic nature of the sticking process.

The sticking ability of the influenza virus may be ascribed to exposed proteins that enable adsorption on the electrode surface.^29^ A solution containing 8 nM silver nanoparticles without the virus produced a negligible stripping charge after 40 min exposure time (red circle), indicating the low tendency of the silver nanoparticles alone to adsorb onto a carbon surface. These results explicitly demonstrate the ability to track *in vitro* a solution containing low concentration of influenza virus (pM range) simply by exploiting its adsorption mannerism together with the inherent electrochemical activity of the silver nanoparticles labels. It is worth mentioning that a solution containing *E. coli* and silver nanoparticles could not be tracked *in vitro* and only after modifying the electrode (by drop casting), could the electroactive properties of the hybrid bacteria/nanoparticles be resolved.^25^ In other words, the adsorption properties of the influenza virus can be exploited here and it has been shown to cause a noticeable striping charge when the solution contains a pM range of the virus, while in the case of ‘tagged’ bacteria direct modification of the electrode is needed, since the bacteria does not adsorbed spontaneously on the carbon electrode.

### Single virus detection *via* ‘nano-impact’

**Fig. 4:**
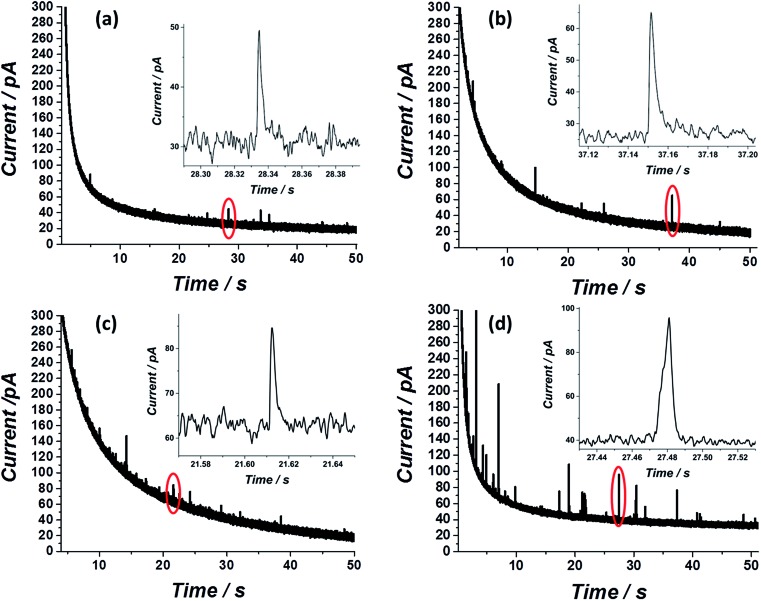
Nano-impacts of individual influenza viruses covered with silver nanoparticles at a carbon microelectrode in a solution containing (a) 0.42 pM influenza virus and 1 nM silver nanoparticles (b) 0.42 pM influenza virus and 2 nM silver nanoparticles (c) 0.84 pM influenza virus and 2 nM silver nanoparticles. (d) 1.26 pM influenza virus and 8 nM silver nanoparticles. For all experiments 0.6 V *vs.* SCE was applied to the working carbon microelectrode.

After showing that the virus can be electrochemically tracked by simply exposing it to silver nanoparticles, the electrochemical properties of the ‘tagged’ virus were then extended to realise detection at the single virus level. This was performed through the use of the nano-impacts technique.^31^,^32^ Recently, a new nano-electrochemical technique has emerged which takes advantage of excluded capacitance contribution *via* chronoamperometric measurements. In brief, by this so called *nano-impacts* technique, random individual collisions of nanometre sized particles can be seen as current spikes or steps in the chronoamperogram, providing that the electrode is held under a sufficient potential to drive an electrochemical reaction such as the oxidation (or reduction) of the nanoparticles. The area under each individual current spike describes the amount of charge oxidised (or reduced) during each collision. By this method, the detection of various individual nanoparticles,^33^–^36^ liposomes^37^–^39^ and organic particles^40^ has been achieved. Very recently this method has also been applied for the detection of biological molecules such as proteins and DNA.^41^,^42^ Using the same principle, we show here that single influenza virus detection can also be realized. A smooth, spikeless chronoamperogram resulted when either the virus or the silver nanoparticles are separately injected into a 0.1 M KCl solution (Fig. S4†). The small nanoparticles themselves contain approximately 25 000 silver atoms, precluding the identification of individual silver nanoparticle impacts as the response is masked by electrical noise. However, when silver nanoparticles are added to a solution containing the virus, clear current spikes are seen in the chronoamperogram (Fig. 4). These spikes are the result of random collisions of individual influenza viruses from a solution containing many viruses, at a carbon microelectrode. The electrode is held at a sufficiently applied oxidizing potential (0.6 V *vs.* SCE) and the random spikes reflect the oxidation of nanoparticles on the ‘tagged’ virus randomly colliding with the electrode. Fig. 4 shows that the number of impacts during a 50 s scan and their magnitude are correlated with the virus concentration and with the concentration of silver nanoparticles, respectively. Specifically, a comparison between Fig. 4a and b shows that a solution with identical viral concentration but with varied silver nanoparticle concentration (*i.e.* the molarity ratio is changed), a similar spike frequency is observed but with a different average current magnitude arising from individual spikes. This observation can be explained by a higher coverage of the nanoparticles on the unsaturated virus surface. Hence, upon an individual collision of a virus with the biased electrode, a higher charge is seen when a higher concentration of silver nanoparticles is used, reflecting a higher number of silver nanoparticles oxidized at each individual collision. The charge magnitude reflects the number of silver nanoparticles that are being oxidized upon virus/electrode impact. In the same manner, comparison of Fig. 4a and c shows that the average number of current transient impacts is increased upon increasing the virus concentration, reflecting the higher probability of random collisions of the virus with the electrode surface. However, the magnitude of the charge on average is conserved given that the virus/silver nanoparticles molarity ratio is kept constant. Lastly, when both the virus concentration and the molarity ratio were increased, the impact frequency and the charge were increased, respectively, as can be seen by comparing Fig. 4a and d.

**Fig. 5:**
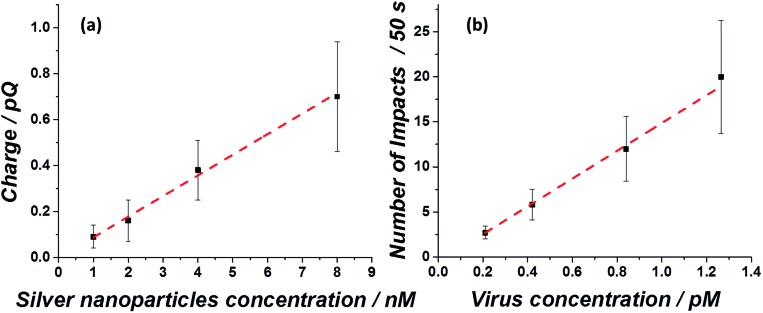
Linear correlation between (a) the average spike charge as a function of silver nanoparticles injected into a solution of 0.84 pM influenza virus and (b) the average number of impacts during a 50 s scan as a function of influenza virus concentration, irrespective of the silver nanoparticle concentration injected.

Both the average charge observed in each spike (Fig. 5a) and the average number of impacts (Fig. 5b), are linearly correlated with the virus/silver nanoparticles molarity ratio and the virus concentration. The errors seen in the observed charge probably reflect different coverage distributions of the silver nanoparticles on the influenza virus surface. These ‘calibration’ curves not only enable the rapid detection of the influenza virus in a given solution at a sub pM level, but also provide a quantitative analysis for the virus concentration, suitable for point of care testing.

### Comparison of the nano-impact technique with existing techniques and bacterial detection

One of the leading techniques for rapid viral and bacterial detection is real time reverse transcription polymerase chain reaction (RT-PCR).^43^*Via* this method, in less than an hour a specific infection can be detected.^44^ RT-PCR uses fluorescent reporter molecules to monitor the production of amplified products during each cycle of the PCR reaction. This combines the nucleic acid amplification and detection steps into one homogeneous assay. Indeed, this method which is nowadays being standardized has the advantage of higher sensitivity, selectivity and duration of the diagnosis over the classical methods used. Still, in many cases the practical solution should more preferably be in the form of a point of care diagnosis, which is at the moment a limitation of the technique. The technique reported here overcomes this issue whilst distinguishing between bacterial and viral components of a solution. It is important to emphasize that although selectivity to specific species is of great need, the main question to be answered in most diagnostic cases is: is the infection viral or bacterial?

From this perspective, an unadorned approach is required. A simple diagnostic tool that can answer this question with a minimal number of components, that can be implemented at the point of care, is at least as important as the ability to selectively detect different species. Moreover, the technique presented here is performed by a nonspecific modification of the nanoparticles and can in principle provide greater selectivity towards sub-species by modifying the electrochemically active nanoparticles with appropriate antibodies or other recognition elements.

Very recently, ‘blocking collisions’ of proteins, DNA and cytomegalovirus^41^,^45^ was demonstrated. In this approach, non-electroactive species can be detected by partially ‘blocking’ an electrode that is electrochemically active to an additional redox species (such as ferrocene) which is added in large quantities to the solution. The method itself has a low signal to noise ratio (even at the high electrolyte concentrations used) and a fabricated nano-electrode, rather than a commercially available microelectrode is needed. In these experiments, amplification (and selectivity) of the detected cytomegalovirus signal was achieved by adding three more components – polystyrene beads and primary and secondary antibodies. In contrast to this approach, in the present paper the signal to noise ratio depends on the amount of silver nanoparticles covering the virus surface. The signal itself arises from the oxidation of the silver nanoparticles and therefore is amplified in comparison to the blocking collisions approach.

The signal arising from a single virus ‘tagged’ with silver nanoparticles is governed by the nanoparticle concentration, and a maximum observed average charge of 0.7 pC can be detected (around 200 silver nanoparticles per virus). Using the same approach for the *in vitro* detection of single *E. coli* ‘tagged’ with silver nanoparticles produced a maximum charge of 9 pC (Fig. S5†). The surface area of *E. coli* bacteria is about 60 fold higher than the virus; therefore, more silver nanoparticles can cover a single bacterium, leading to a higher signal due to the higher number of silver nanoparticles being oxidized.

## Conclusions

Here, we show that nanoparticles themselves can serve as a detected element, as an integrated part of the virus species upon interaction. By this approach, two important goals are achieved: (a) the electrochemical signal detected is enhanced, and (b) commercially available microelectrodes are sufficient for detection purposes. We have shown here by UV-Vis characterization that upon the interaction of silver nanoparticles with the bacteria or virus, the plasmonic peak is maintained, even at high ionic strength. However, by UV-Vis spectroscopy the two species are indistinguishable (Fig. 1). Exploiting the electrochemical property of the ‘tagged’ biological species reveals the differences between the bacteria and the virus. At first, the ability of the ‘tagged’ virus to adsorb on a carbon electrode is seen (a property absent in the case of bacteria) and this may be related to the biological function of the virus (where exposed proteins serve as sticking elements). On a physical level, the fact that viruses in general are at least an order of magnitude smaller than bacteria can be manifested in the signal arising from individual species. A further step towards the utilization of this technique may be *via* the use of modified nanoparticles that can bind only to a specific organism. These kinds of modifications have been established already for various species such as streptavidin modified magnetic nanoparticles for the detection of the hepatitis B virus,^46^ poly(hexamethylene biguanide) functionalized magnetite nanoparticles for *E. coli* detection and separation,^47^ antibody functionalized gold nanoparticles for influenza virus detection,^48^ functionalized nanoparticles conjugated to monoclonal antibodies for detecting respiratory syncytial,^49^*etc.*

Lastly, commercially available microfluidic electrochemical sensors are now reaching the sub nA noise level and a further decrease in the noise level is more of a low demand issue rather than an electronic obstacle. Consequently, automated microfluidic electrochemical sensors combined with the proposed technique may be designed for point of care testing of viral and bacterial infections as a standard diagnostic tool.

In summary, we have applied here a state of the art electrochemical technique used mostly for the detection and characterization of a low concentration of nanoparticles in solution, and broadened its application to demonstrate fast, cost effective and easy to use detection of the influenza virus in solution.

## Materials and methods

Silver nanoparticles of 9.4 ± 3.6 nm were synthesized according to the literature^23^ and as described in the ESI.† Sodium nitrate (>99.5%, NaNO_3_) was supplied by Fisons Scientific Equipment, Loughborough, UK. Trisodium citrate (>99%, Na_3_C_6_H_5_O_7_) was ordered from BDH Laboratory Supplies, Poole, UK. Silver nitrate (>99%, AgNO_3_) and potassium chloride were obtained from Sigma-Aldrich, Dorset, UK. Sodium borohydride (99%, NaBH_4_) was supplied by Fisher Scientific, Loughborough, UK. The influenza A virus (H1N1), A/PR/8/34, was purchased from LGC ATCC (ATCC-VR-95) and experiments were done in a biosafety level 2 authorized environment. All solutions were made with ultrapure water from Millipore with a resistivity of not less than 18.2 MΩ cm at 298 K.

The characterization of the silver nanoparticle suspension with and without the influenza virus was performed by UV-Vis spectroscopy (1800 UV, Shimadzu). For all measurements a quartz cell with a 1 cm optical path was used.

For the electrochemical measurements of the ‘sticky’ virus, a 7 μm diameter carbon fibre wire (Goodfellow Cambridge Ltd.), of approximately 1 mm in length was used as a working electrode, as reported earlier.^50^ Experiments were conducted at 293 ± 2 K within a Faraday cage by using a saturated calomel reference electrode (SCE) and a graphite rod counter electrode. For the chronoamperometric measurements, a homemade potentiostat was used together with a micro carbon disk electrode (diameter = 33 μm). A full description of the system is provided elsewhere.^35^ A 4 kHz preamplifier was used and filtered with a built-in passive 100 Hz filter. Impact spikes were analysed using the Origin v.8.1 program (; http://www.OriginLab.com) and SignalCounter software developed by Dario Omanovic (Centre for Marine and Environmental Research, Ruder Boskovic Institute, Croatia).

Transmission electron microscopy was performed using a JEOL 3000F instrument operated at 300 kV. Sample preparation consisted of modifying holey carbon grids by drop casting suspensions of the nanoparticles, viruses or nanoparticle-modified viruses and allowing them to dry. It should be noted that no stain was used during this imaging. Energy dispersive X-ray mapping was performed using an Oxford Instruments Si(Li) detector running the INCA processor, *via* the scanning transmission electron microscopy (STEM) mode. Here the electron probe was limited to a diameter of *c.a.* 1 nm and scattered electrons were collected with a high-angle annular dark field (HAADF) detector with inner angle of 50 mrad, giving highly Z-contrast imaging.

## Supplementary Material

Supplementary information
