# Supplementary material for: Rapid electrochemical detection of single influenza viruses tagged with silver nanoparticles
Source: Chem Sci. 2016 Feb 25;7(6):3892–9. doi: 10.1039/c6sc00412a (PMC6013776; doi:10.1039/c6sc00412a)
Supplement: Supplementary file 1 [file SC-007-C6SC00412A-s001.pdf]

Electronic Supplementary Material (ESI) for Chemical Science.
This journal is © The Royal Society of Chemistry 2016

Supplementary Information

Rapid Electrochemical Detection of Single Influenza Virus Tagged with Silver Nanoparticles

Lior Sepunaru[a], Blake J. Plowman[a], Stanislav V. Sokolov[a], Neil P. Young[b] and Richard G. Compton[a,1]

a. Physical and Theoretical Chemistry Laboratory, Department of Chemistry, University of Oxford, Oxford OX1 3QZ, United Kingdom

b. Department of Materials, University of Oxford, Oxford OX1 3PH, United Kingdom

[1] To whom correspondence should be addressed. Email: richard.compton@chem.ox.ac.uk

Nanoparticle tracking analysis was employed for characterization of the influenza virus concentration in solution and for the average virus size. The video files of the Brownian motion were analysed using a Nanosight instrument (642 nm) fitted with NTA 2.3 software and the observed mean size of the influenza virus was 125 nm with a multiplicative standard deviation of 0.5, in agreement with previous results using the same technique[1]. The virus concentration was estimated using the same techniques by integrating the number of total counts arising from various dilution of the stock solution.

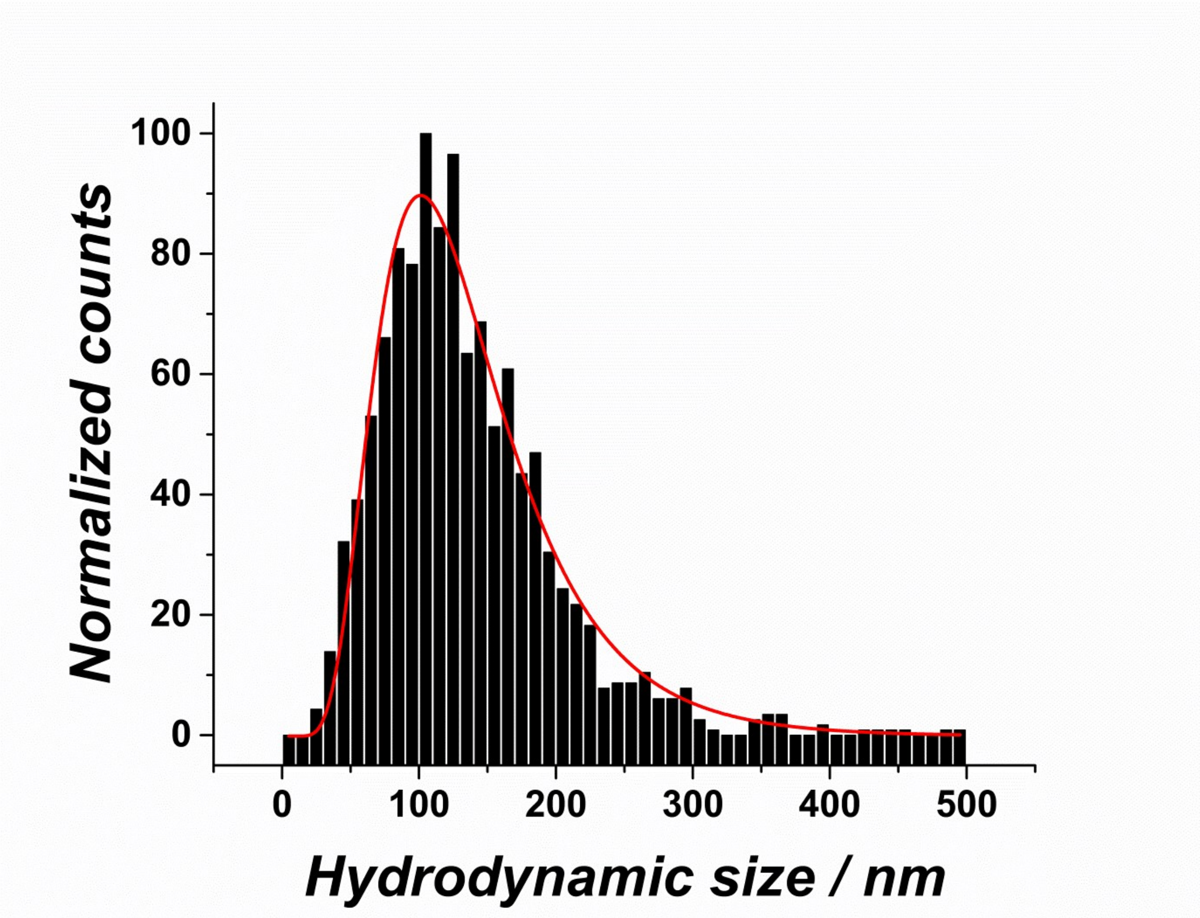

Fig. S1 Nanoparticles tracking analysis of the hydrodynamic diameter of influenza virus in water.

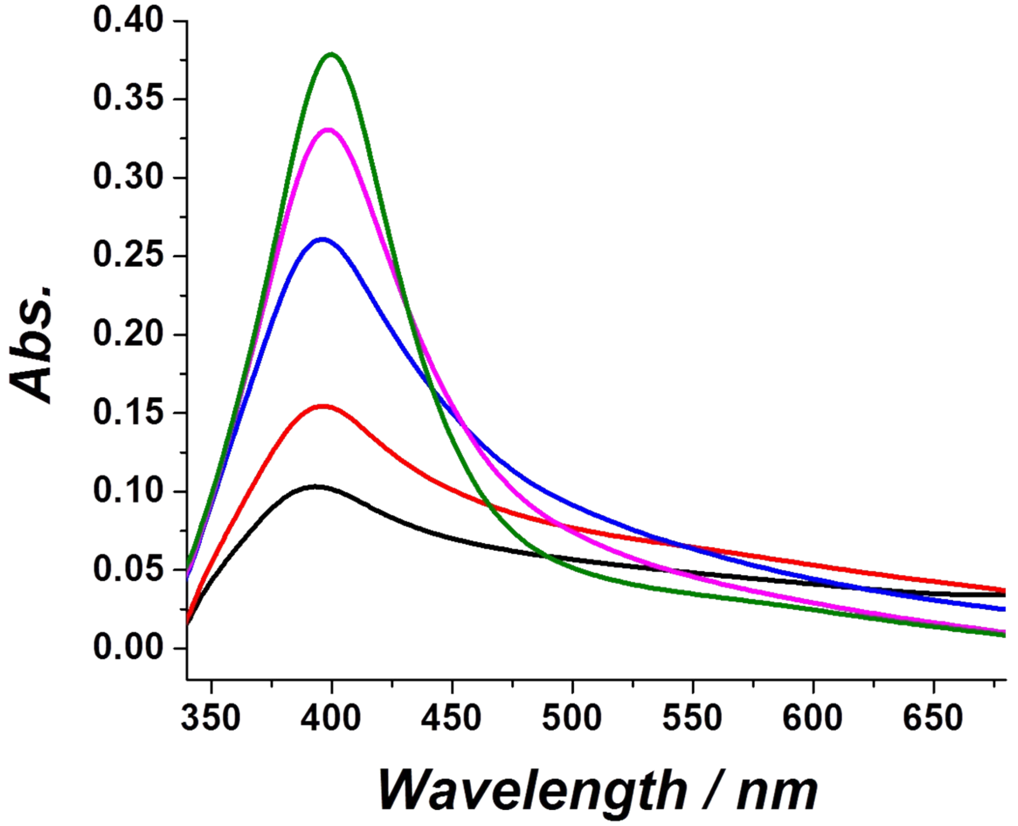

Fig. S2. Absorption spectra of 1 nM silver nanoparticles injected into a solution containing various concentration of influenza virus in 0.1 M KCl solution. The absorption peak signal from highest to lowest: >0.125 pM, 0.625 pM, 312 fM, 156 fM, 78 fM of influenza virus.

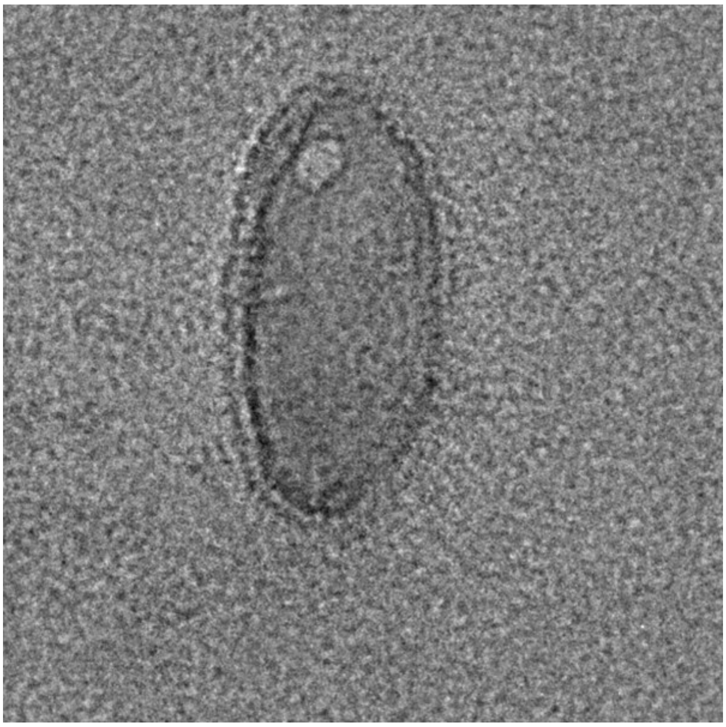

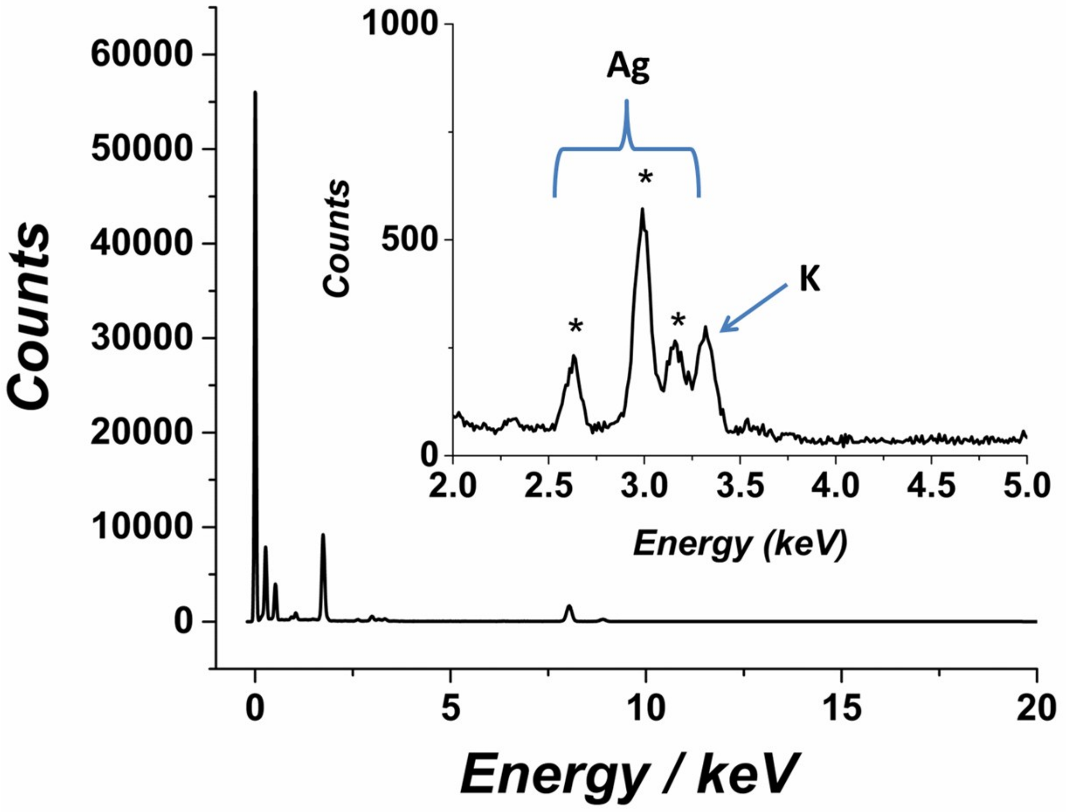

Fig. S3 (Top) TEM image of an unstained influenza virus. (Bottom) EDX spectra of the virus decorated with silver nanoparticles, as shown in Fig. 2d. (Inset) silver characteristics peaks (and potassium) arising from the nanoparticle decorated virus.

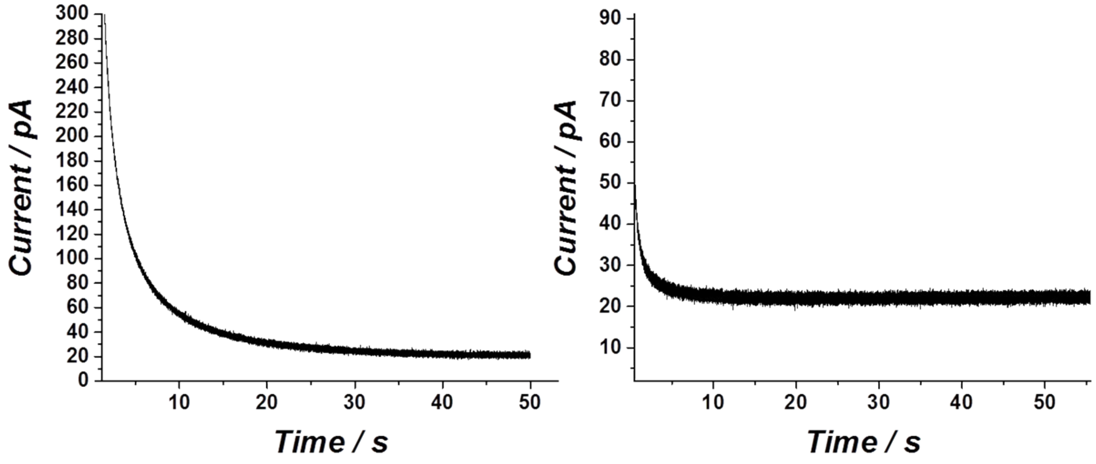

Fig. S4. Nano-impacts of (a) 0.84 pM influenza virus in 0.1 M KCl (b) 8 nM of silver nanoparticles in 0.1 M KCl. 0.6 V vs SCE was applied to the working carbon microelectrode.

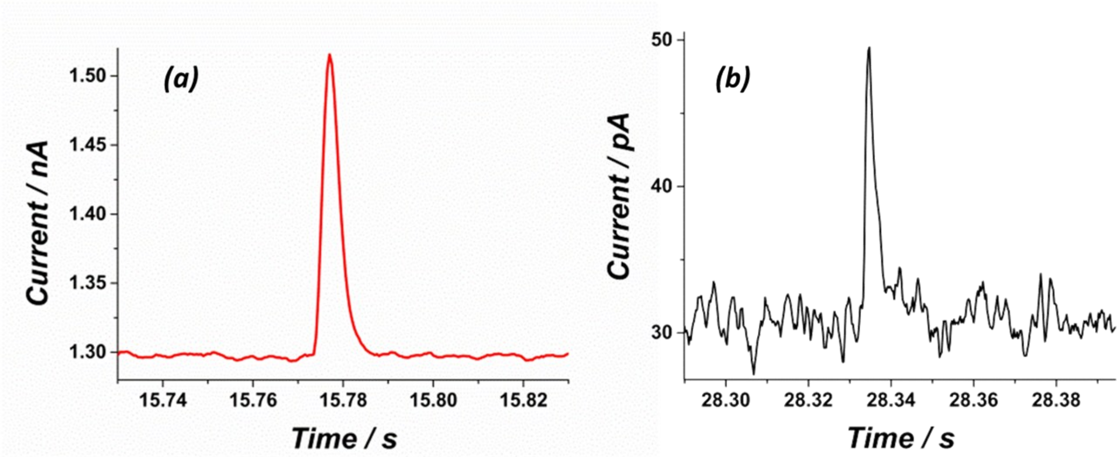

Fig S5. Representative current spikes of an impacting E. coli (a) and influenza virus(b) exposed to 1 nM silver nanoparticles. The current spike magnitude of E. Coli is 200 pA while for the influenza virus it is 20 pA.

References:

1 P. Kramberger, M. Ciringer, A. Štrancar and M. Peterka, *Virol. J.*, 2012, **9**, 265.
